# Multi-compartment Microfluidic Device Geometry and Covalently Bound Poly-D-Lysine Influence Neuronal Maturation

**DOI:** 10.3389/fbioe.2019.00084

**Published:** 2019-05-07

**Authors:** Joyce W. Kamande, Tharkika Nagendran, Joseph Harris, Anne Marion Taylor

**Affiliations:** ^1^UNC/NC State Joint Department of Biomedical Engineering, The University of North Carolina at Chapel Hill, Chapel Hill, NC, United States; ^2^UNC Neuroscience Center, The University of North Carolina at Chapel Hill, Chapel Hill, NC, United States; ^3^Xona Microfluidics, LLC, Temecula, CA, United States

**Keywords:** neuron, human stem cells, standard neuron device, multi-compartment devices, microfluidic chambers, silicone chambers, glutamatergic neurons, compartmentalized microfluidic platform

## Abstract

Multi-compartment microfluidic devices have become valuable tools for experimental neuroscientists, improving the organization of neurons and access to their distinct subcellular microenvironments for measurements and manipulations. While murine neurons are extensively used within these devices, there is a growing need to culture and maintain human neurons differentiated from stem cells within multi-compartment devices. Human neuron cultures have different metabolic demands and require longer culture times to achieve synaptic maturation. We tested different channel heights (100 μm, 400 μm, and open) to determine whether greater exposure to media for nutrient exchange might improve long-term growth of NIH-approved H9 embryonic stem cells differentiated into glutamatergic neurons. Our data showed an opposite result with both closed channel configurations having greater synaptic maturation compared to the open compartment configuration. These data suggest that restricted microenvironments surrounding neurons improve growth and maturation of neurons. We next tested whether covalently bound poly-D-lysine (PDL) might improve growth and maturation of these neurons as somata tend to cluster together on PDL adsorbed surfaces after long culture periods (>30 days). We found that covalently bound PDL greatly improved the differentiation and maturation of stem cell-derived neurons within the devices. Lastly, experimental paradigms using the multi-compartment platform show that axons of human stem cell derived neurons intrinsically regenerate in the absence of inhibitory cues and that isolated axons form presynaptic terminals when presented with synaptic targets.

## Introduction

Microfluidic devices offer useful platforms for neuroscience that can mimic the neuron microenvironment on a cellular level. This is important for mechanistic studies, such as those pertaining to the pathology of neurodegenerative disorders (Poon et al., 2011; Wu et al., 2013; Zala et al., 2013; Van Laar et al., 2018; Zhang et al., 2018) and neurotrauma (Nagendran et al., 2017; Ohtake et al., 2018). Multi-compartment microfluidic devices provide enhanced accessibility to neuronal microenvironments, which would otherwise be arduous in conventional formats due to stochastic orientations of axons and dendrites (Taylor et al., 2005, 2010). Historically, these devices are made using optically transparent and biocompatible poly(dimethylsiloxaone) (PDMS) either by researchers directly or purchased commercially. The neuroscience community has enthusiastically adopted these devices in recent years (Neto et al., 2016) and these devices have become essential tools for hundreds of labs world-wide. Multi-compartment devices are configured to fluidically isolate distinct segments of neurons of cell bodies, dendrites, axons, and synapses. Consequently, this enables manipulation of these segments as well as visualization using high resolution live imaging. Studies have been achieved that would not have been feasible *in vivo* using these microfluidic platforms, such as studies of axonal transport, biochemical analysis of axons and axonal injury/regeneration [e.g., Taylor et al., 2005, 2013; Poon et al., 2011, 2013; Wu et al., 2013; Zala et al., 2013; Bigler et al., 2017; Nagendran et al., 2017].

The majority of neuron based culture platforms use animal model neurons such as rat or mouse neurons as surrogates for human neurons. Animal neurons have some innate qualities that do not make them ideal representative models for studies of the human CNS. For example, differences in genetic and morphological make up. There is mounting interest in using human stem cell (hSC) derived neurons for human disease models and cell-based high throughput screens (Farkhondeh et al., 2019). Nevertheless, there still lies some challenges in the culture of hSC-derived neurons since they are difficult to maintain, variable, and require longer maturation times than murine neurons (Niedringhaus et al., 2015). Survival of hSC-derived neurons is also influenced by nutrient depletion, cell density, extracellular matrix (ECM) substrate stability, and shear force from fluid movement (Bigler et al., 2017).

To improve long-term growth of hSC-derived neurons, three different configurations of PDMS based compartmentalized devices were evaluated. Two of the configurations fall under a closed compartment format while the other is an open compartment format. Specifically, the two closed configurations vary in channel height leading to differences in shear force due to fluid movement within the channels. We also evaluated different ECM substrates as well as their stability on these devices for long-term culture (>30 DIV) of the cells using different attachment assays. Finally, we demonstrated assays using microfluidic devices with hSC-derived neurons, including assays for axon injury/regeneration and synapse development.

## Material and Methods

### Materials List

Sylgard® 184 Silicone elastomer Kit from Dow Corning (Midland, MI); APTS (3-Aminopropyltriethoxysilane) from Sigma Aldrich (St. Louis, MO); BS^3^ (Bis (sulfosuccinimiidyl) suberate) from Pierce Biotechnology (Rockford, IL): PDL (Poly-D-Lysine) from BD Biosciences (San Jose, CA); propidium Iodide from Molecular probes (Grand Island NY); CellTracker™ Green CMFDA Dye from Molecular probes (Grand Island NY); primary Chicken antipeptide β-Tubulin III from Aves Labs (Tigard, OR); and, DAPI from Sigma Aldrich (St. Louis, MO).

### SU8-Si Master Fabrication

SU8 on Si master fabrication has been reported elsewhere (Taylor et al., 2003, 2005). Masks to generate the SU8-Si masters were drafted in AutoCAD (Autodesk Inc.) and chrome masks were generated (Photo Sciences, Inc.). Masters were fabricated in the Chapel Hill Analytical & Nanofabrication Laboratory (CHANL) at UNC. SU8-2005 (Microchem) was spun onto a silicon wafer to generate the 4 μm layer. SU8-2050 was spun on at a thickness of 120 μm, for the 400 μm tall chamber, SU8 laminates of thickness 500 μm were embossed onto the 4 μm layer.

### Multi-compartment Device Preparation

Poly(dimethylsiloxane) (PDMS) was molded onto a SU-8 master, as described previously (Taylor et al., 2003, 2005). Open chambers were provided by Xona Microfluidics, LLC. Devices were sterilized in 70% ethanol and placed onto PDL coated glass coverslip substrates as described previously. We used 500–550 kDa PDL for both covalent and adsorbed conditions. For adsorbed conditions, we incubated glass for >6 h at 37°C.

For chambers with covalently bound PDL, glass was first exposed to 90 W oxygen plasma for 5 min to generate hydroxyl functionalities on the glass surface. Next, amino groups were generated via overnight vapor deposition of APTS. PDL was then covalently attached using a bifunctional crosslinker BS^3^. Wettability was evaluated using contact angle measurements.

### H9 Culture

The NIH-approved, human embryonic stem cell (ESC) line H9 (WA09) was obtained from WiCell Research Institute (Madison, WI). Cells were maintained and differentiated as described previously (Zeng et al., 2010; Niedringhaus et al., 2015; Bigler et al., 2017). H9 ESCs were maintained as undifferentiated colonies on growth factor reduced Matrigel (BD Biosciences) in mTeSR1 media (StemCell Technologies). Media was changed daily. Cells were passaged every 3 days with 0.5 mM EDTA (340 mOs).

After 24 days *in vitro* (DIV) of culturing H9 human embryonic stem cells, neural rosettes, which are formed from differentiated neural progenitor cells (NPCs), were dissociated and harvested from poly-L-ornithine/Laminin coated plates and seeded into the 3 configurations of multi-compartment devices at equivalent densities. Once the NPCs attached within the microdevices they were maintained for further differentiation and maturation (Bigler et al., 2017).

### Mcherry Virus Infection

G-deleted Rabies-mCherry virus (Wickersham et al., 2007) (Salk Institute; 1 × 10^5^ viral units) diluted in 50 μl-conditioned media was added to the axonal compartment of each chamber and incubated for 2 h at 37°C. Conditioned media without virus was added back to the axonal compartments following two washes with fresh media. Devices were maintained in 37°C incubator for ~48 h until mCherry expression was visible.

### Synapse Forming Beads

Carboxylated 6 μm beads were covalently conjugated with PDL using 200 mg EDC/2 mg NHS in MES buffer pH 5.5. PDL beads were then added to the axonal compartment and incubated for 3 h or overnight for synaptic vesicle clustering.

### Immunocytochemistry

Neuron cultures were fixed with PFA and permeabilized in 0.25% Triton X-100. Cultures were then blocked in 10% normal goat serum for 15 min. Coverslips were incubated with anti-Map2 (1:1,000; Millipore and Sigma), anti-Synapsin-1 (1:500; Calbiochem), anti-βTubulinIII (1:2,000; Aves labs) and/or anti-vGLUT1 (1:100; NeuroMab) primary antibodies in 1% blocking solution for overnight at 4°C. Coverslips were then incubated with goat anti-mouse or anti-rabbit or anti-chicken secondary antibodies conjugated to Alexa-fluorophores (1:1,000; Invitrogen) for 1 h at RT. Coverslips were then rinsed once using PBS and counterstained with DAPI in 1 × PBS for 5 min at RT. Following PBS washes coverslips were mounted onto the glass slide.

### FM Unloading

Neurons were loaded with lipophilic dye N-(3-trimethylammoniumpropyl)−4-(6-(4-(diethylamino) phenyl)hexatrienyl)pyridinium dibromide (FM 5–95; Invitrogen) using KCl mediated depolarization (Taylor et al., 2013). Cultures were first incubated for 30 min with pre-warmed HEPES-buffered solution (HBS; 119 mM NaCl, 5 mM KCl, 2 mM CaCl_2_, 2 mM MgCl_2_, 30 mM glucose, and 10 mM HEPES). Media was then replaced with FM dye loading solution containing 10 μM FM 5–95, 20 μM AMPAR antagonist 6-cyano-7-nitroquinoxaline-2,3-dione disodium (CNQX; Tocris), 50 μM NMDAR antagonist D-(-)-2-amino-5-phosphonopentanoic acid (D-AP5; Tocris) in 90 mM KCl HBS for 1 min. The loading solution was replaced with HBS containing 10 μM FM 5–95 for 1 min and later rinsed three times with a high-Mg^2+^, low-Ca^2+^ solution (106 mM NaCl, 5 mM KCl, 0.5 mM CaCl_2_, 10 mM MgCl_2_, 30 mM glucose, and 10 mM HEPES) containing 1 mM Advasep-7 (Biotium) to remove extracellular membrane-bound FM. Finally, cultures were washed in HBS containing 20 μM CNQX and 50 μM D-AP5 for at least three times, 1 min each. Next, we stimulated the microfluidic chambers using extracellular electrodes by placing a positive and negative electrode in each well of the somatodendritic compartment.

Electrical stimulation was provided by an AD Instrument 2 Channel Stimulus Generator (STG4002) in current mode with an asymmetric waveform (−480 μA for 1 ms and + 1,600 μA for 0.3 ms) for ~1 min at 20 Hz for 600 pulses. The FM 5–95 imaging was performed using a spinning disk confocal imaging system^24^. Z-stacks (31 slices) were captured every 15 s during the baseline (1 min), stimulation (1 min), and after stimulation (2 min) periods. At least 3 baseline images were acquired before electrical stimulation.

### Microscopy

FM and fixed imaging was performed using CSU-X1 (Yokogawa) spinning disk confocal imaging unit configured for an Olympus IX81 microscope (Andor Revolution XD). Excitation for the spinning disk confocal imaging system was provided by 405, 488, 561, and/or 640 nm lasers. The following bandpass emission filters (BrightLine, Semrock) were used for the spinning disk: 447/60 nm (TRF447-060), 525/30 nm (TRF525-030), 607/36 nm (TR-F607-036), and 685/40 nm (TR-F685-040). For FM imaging, the spinning disk confocal imaging system was used with excitation at 561 nm and the 685/40 nm emission filter. We used 2 × 2 binning to reduce the laser intensity and acquisition time for each frame; each z-stack was obtained in ~5 s.

### Statistical Analysis

All statistical analyses were performed using GraphPad Prism. The statistical test, sample size, and *p*-values are described in the figure legends or in the results section for contact angle measurements.

## Results

### Open and Closed Configurations for Culturing hSC-Derived Neurons

**Figure 1:**
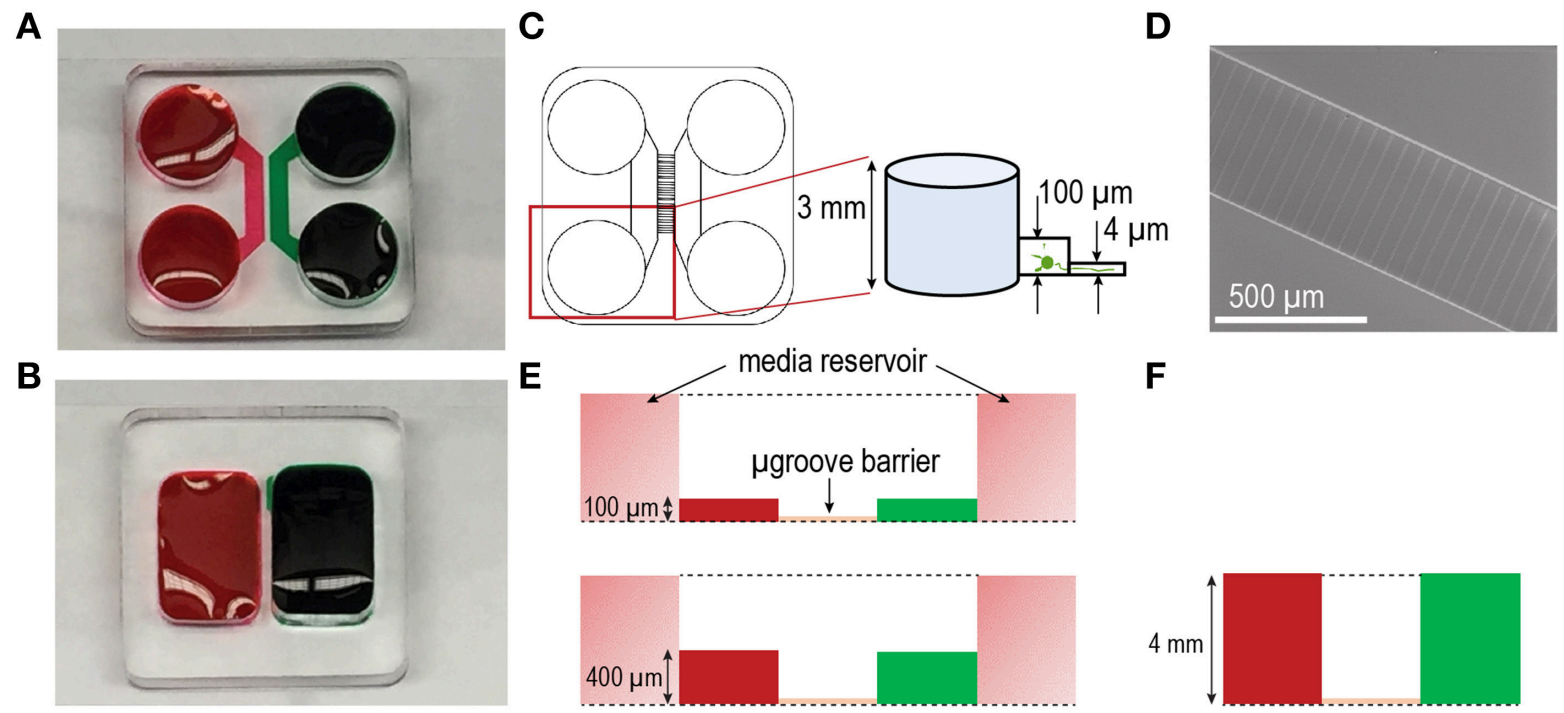
Multi-compartment device configurations for culturing human stem cell derived neurons. Photographs of **(A)** closed and **(B)** open multi-compartment device configurations. Compartmentalization within these devices allow for axonal isolation from somata. Fluidic isolation (demonstrated with red and green food coloring dyes) within the devices is achieved via microgroove-embedded barriers. **(C)** Approximately 150 microgrooves separate each compartment. The inset shows feature heights of the standard closed configuration device including a compartment height of ~100 μm and microgroove height of ~4 μm. In the closed channel configuration each compartment is flanked by 2 media reservoirs ~3 mm in height. **(D)** SEM image of the microgroove region in the SU-8 photoresist-patterned master used to mold PDMS devices via soft lithography. The width of each microgroove is ~10 μm. **(E)** Diagram of the closed channel configurations differing in compartment height ~100 μm (above) and ~400 μm (below). **(F)** Diagram of the open chamber configuration with compartment height of ~4 mm.

Closed channel PDMS based multi-compartment devices for CNS neuronal cultures were first described by Taylor et al. (2005). The purpose of these devices was to provide accessibility to the different compartments (somata and axons) of neurons *in vitro* to facilitate axon and synapse centered biological studies mainly using embryonic rodent neurons. These devices consist of two compartments that are fluidically isolated by a microgroove-embedded barrier (Figure 1) (Taylor et al., 2005). The somata and axon compartments in these devices are ~100 μm in height and 1.5 mm in width.

More recently, interests have focused on the culture of hSC-derived neurons as a more representative model for studies of human neurodegenerative diseases as these cells more closely resemble those found in the human brain tissue. However, these cells have different metabolic demands and require longer maturation times than rodent neurons. Therefore, we modified the closed channel multi-compartment configuration raising the height to 400 μm to increase the volume of media immediately surrounding the neurons with the goal of improving nutrient replenishment and resulting cell viability and growth (Figure 1E). By increasing the height to 400 μm, we also increased the linear velocity of the media during media changes due to a reduction in fluidic resistance that occurs as the volume to surface area ratio of the channel becomes lower. Our third multi-compartment configuration consisted of an open compartment device format (Figures 1B,F) where the cell compartments were not enclosed within a channel. This open format provided an increased volume of media and without involving fluid flow through microfluidic channels.

### Differentiation of hSCs Into Neurons Within Multi-compartment Devices

**Figure 2:**
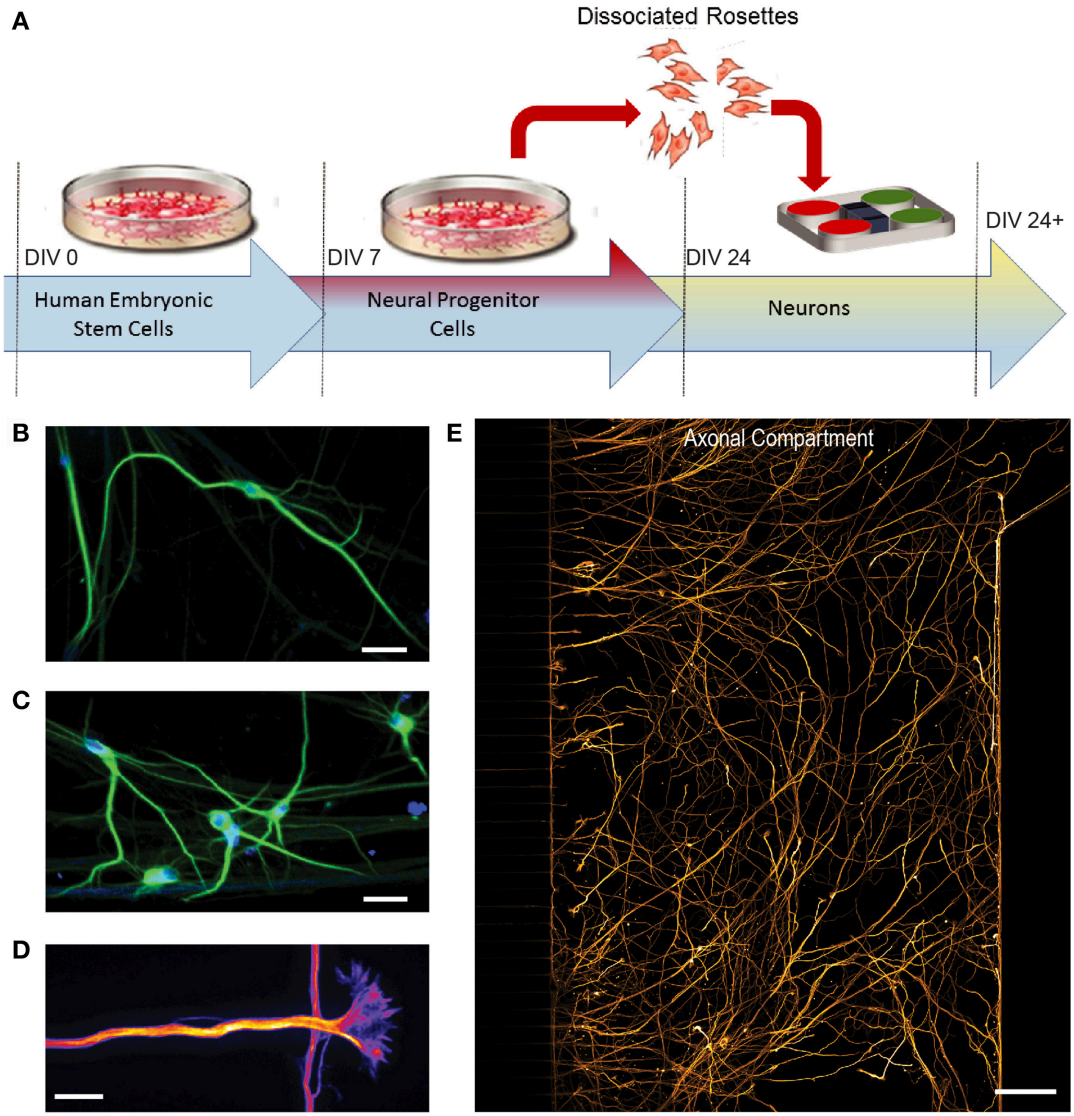
Differentiation of human embryonic stem cells (hSCs) into a glutamatergic neurons. **(A)** H9 hSCs were expanded for 7 days *in vitro* (DIV) and then differentiated into neural progenitor cells (NPCs). After day 24, cells were dissociated from NPC rosettes and seeded into multi-compartment devices. **(B C)** Immunofluorescence images show staining of neuron and dendrite-specific marker MAP2 (green) at 41 DIV. DAPI (blue) labels nuclei. Scale bars, 20 μm. **(D)** Large growth cones, which constitute the distal tip of axons for growth and navigation, were observed by β-Tubulin III immunostaining (Fire LUT). Scale bar, 20 μm. **(E)** β-Tubulin III immunostaining (orange) labels axons in the axonal compartment. Scale bar, 100 μm.

Differentiation of hSCs into post-mitotic neurons began at 24 days *in vitro* (DIV) following expansion of hSCs (Figure 2A). To characterize the hSC-derived neurons within the devices, we used specific neuronal markers to identify the dendritic and axonal compartments of the neuron. Cells immunolabeled with neuron-specific marker MAP2 (microtubule associated protein 2) within the cell compartment of microfluidic devices, demonstrate neuronal differentiation (Figures 2B,C). Large growth cones were also observed extending from neurites through the microgrooves (Figure 2D). Long projections were immunolabeled with neuron-specific marker β-Tubulin III and extended extensively within the axonal compartment (Figure 2E), demonstrating that the hSC cell differentiated into highly polarized neurons. We also analyzed the extent of neuronal differentiation in the three configurations by quantifying the percentage of β-Tubulin III expressing cells to the total population of cells within the compartments counted using the nuclear marker, DAPI. We found that at DIV 24, all configuration showed >75% neuronal differentiation.

### Synapse Maturation of hSC Derived Neurons Within Multi-compartment Devices

**Figure 3:**
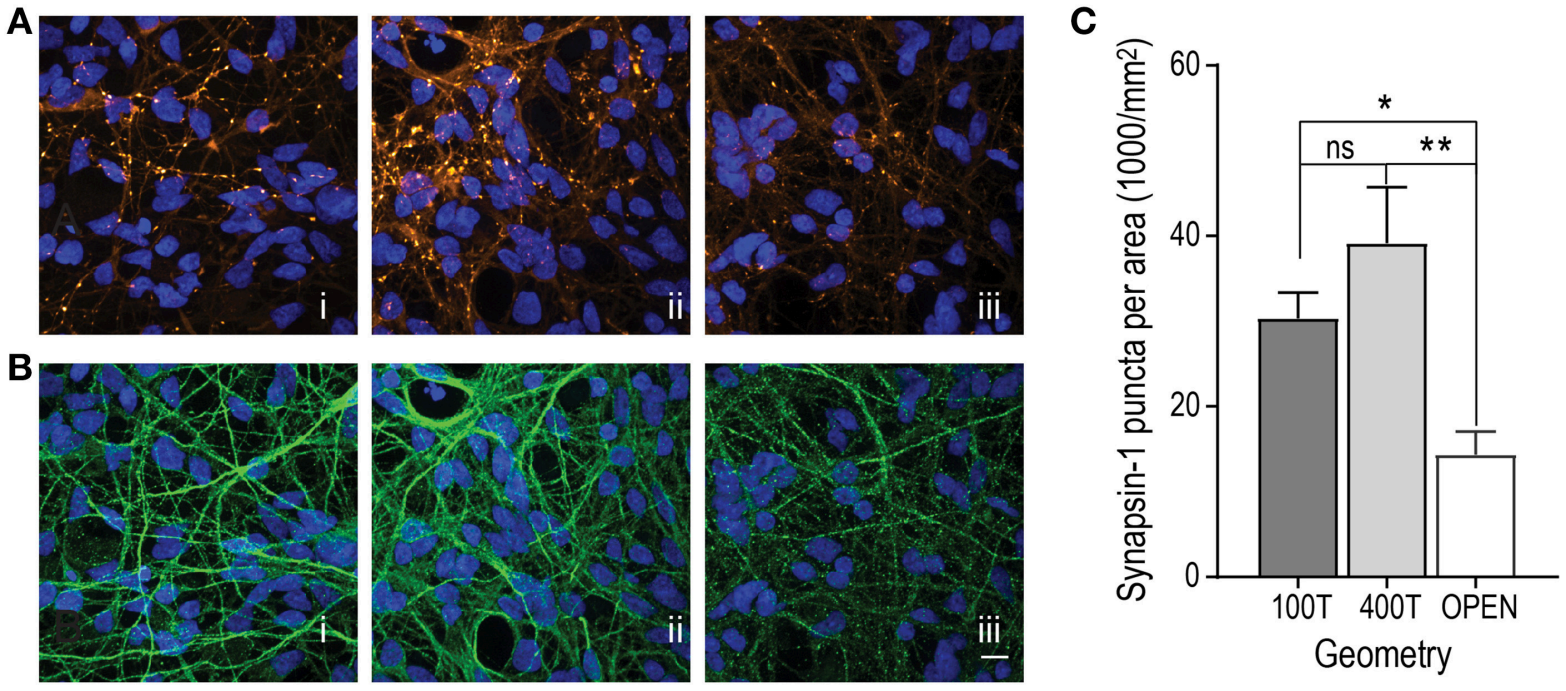
Evaluation of synapse maturation within the 3 multi-compartment chamber configurations [(i) closed chamber 100 μm tall (100T), (ii) closed chamber 400 μm tall (400T), and (iii) open chamber)]. **(A)** Representative fluorescence images of immunolabeled hSC neurons at DIV 34 for synapsin-1 (orange LUT). All nuclei were stained with DAPI (blue LUT). **(B)** Representative fluorescence images of immunolabeled hSC neuronal processes with β-Tubulin III (green LUT). Scale bar, 20 μm. **(C)** Quantification of the mean number of Synapsin 1 puncta per unit neuron area determined by β-Tubulin III immunofluorescence combined from cultures 24 DIV and older. 100T: *n* = 40 frames over 5 devices; 400T: *n* = 36 frames over 4 devices; open: *n* = 28 frames over 3 devices. One-way ANOVA, Tukey's multiple comparisons test, ^*^p < 0.05, ^**^p < 0.01. Error bars, s.e.m.

As neurons mature during development, the number of synapses per neuron increases. To assess the synapse maturation of hSC neurons within the multi-compartment devices, we quantified the number of synapses per neuron area in all three configurations to determine whether one configuration type might improve synapse maturation. We performed immunostaining for synapsin 1, a synaptic vesicle protein associated with mature synapses, and quantified the number of fluorescent puncta per neuron area, immunolabeled with β-tubulin III (Figure 3). Our data show that both closed channel compartment devices, the 100 and 400 μm (trending to be the highest), had significantly higher synapsin 1 density than the open channel device (Figure 3C).

### Poly-D-Lysine Substrate Attachment Strategy and Effect on Neuron Differentiation and Synapse Maturation

Previous reports demonstrated that covalent attachment of poly-D-Lysine (PDL) on a substrate for *in vitro* rodent neuron culture enhances neuron anchorage due to the adhesion stability (Kim et al., 2011). hSC derived neurons require longer culture times in order to reach maturity than rodent cultures; therefore, we hypothesized that covalently bonded PDL substrates would provide more stable substrates for these neurons to grow within the compartmentalized devices and subsequently improve differentiation and synapse maturation.

**Figure 4:**
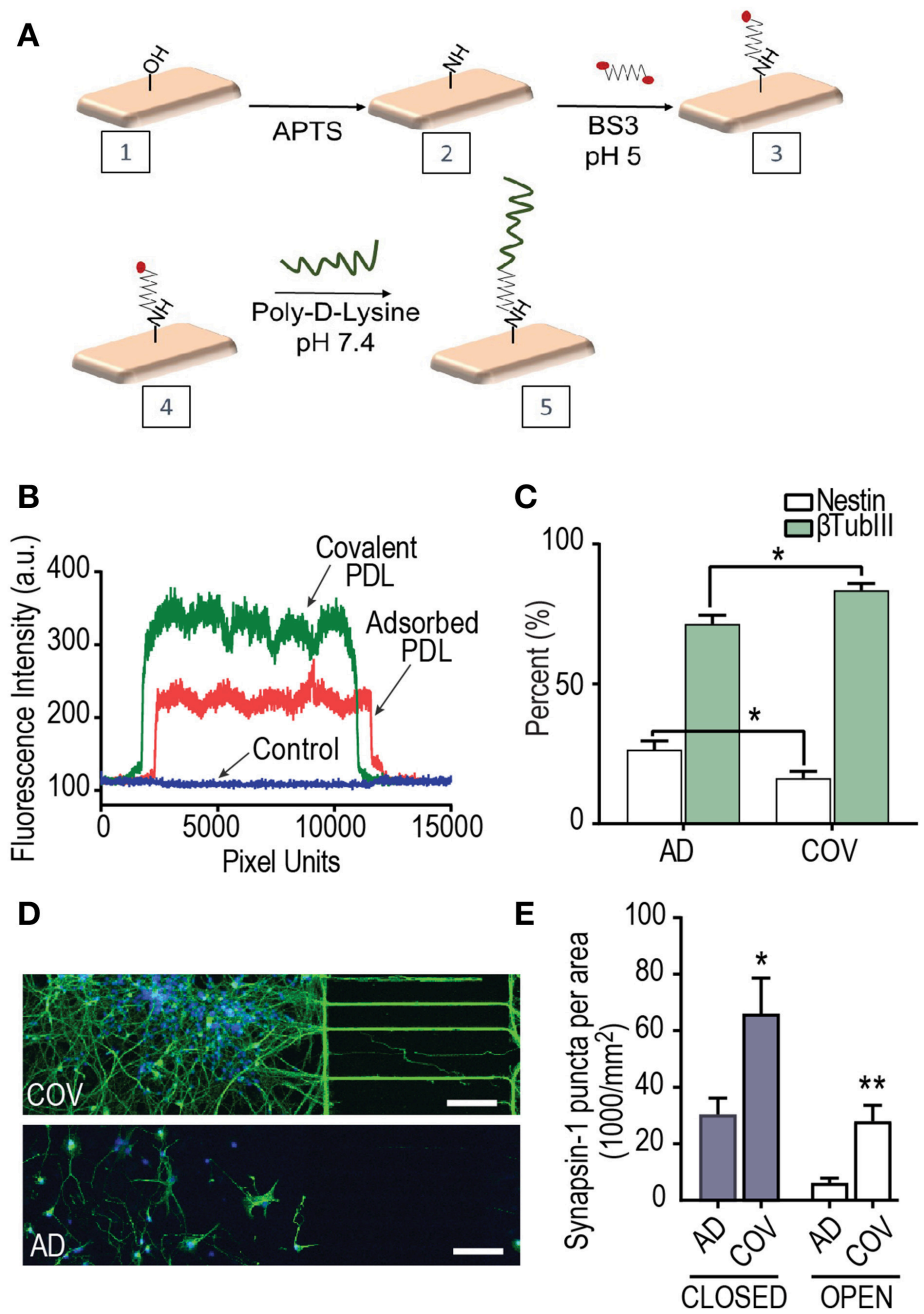
The effect of Poly-D-Lysine (PDL) substrate immobilization on hSC neuron differentiation and synapse maturation. **(A)** Covalent attachment scheme of PDL onto glass coverslips on which neurons attach within multi-compartment devices. Hydroxyl functionalities on the glass surface were generated by oxygen plasma. Overnight vapor deposition of APTS produced surface amino groups. PDL was then covalently attached using a bifunctional crosslinker BS^3^. **(B)** Representative fluorescence intensity plot profiles of FITC-polylysine immobilized onto glass coverslips using covalent and adsorbed strategies. **(C)** Quantification of the percent differentiation of hSCs expressing the neuron marker, β-Tubulin III on covalent (COV) and adsorbed (AD) PDL substrates over DIV 24–34 in 400T multi-compartment devices. The percent of the neural progenitor marker, nestin, was significantly lower when PDL was covalently bound. Unpaired *t*-test for nestin and βtubIII results; adsorbed: *n* = 27 frames over 2 replicates; covalent: *n* = 18 frames over 2 replicates. **(D)** Fluorescence images of hSC neurons at DIV 34 within devices that had covalent and adsorbed PDL, respectively. Neurons were immunostained for βTubIII (green) and nuclei stained with DAPI (blue). Scale bars, 100 μm. **(E)** Quantification of the number of synapsin 1 puncta per neuron area analyzed by immunofluorescence in closed and open multi-compartment devices with adsorbed and covalent PDL substrates. Neuron area was measured by βTubIII immunofluorescence. Unpaired 2-tailed *t*-test; closed AD and COV (*n* = 16 frames each condition over 2 replicates); open AD and COV (*n* = 9 frames each condition). ^*^p < 0.05, ^**^p < 0.01; error bars, s.e.m.

Covalent PDL was bound to the glass surface using the bifunctional linker, BS^3^ (Figure 4A). Both covalent and adsorbed PDL coated surfaces were characterized by fluorescence using a FITC conjugated polylysine and contact angle measurements. The density of PDL was greater along fluorescence line scans as reflected in higher fluorescence intensity values of FITC-polylysine (Figure 4B). Contact angle reflects the wettability of the surface with lower contact angles more stable surface coating. The covalent surface had significantly higher wettability of 17 ± 0.3° (*n* = 9 measurements over 3 samples) than adsorbed surface at 28.72 ± 0.5° (*n* = 9 measurements over 3 samples) (*p* < 0.0001).

We proceeded to compare the effect of differentiation and synapse maturation on the hSC derived neurons within devices that had either PDL covalently bonded (Figure 4A) or PDL substrate physically adsorbed onto the glass coverslips. We quantified the percentage of βTubIII expressing cells to the total population of cells labeled with DAPI within the sets of 400 μm tall devices. Our data show a significant increase in βTubIII expressing cells in devices with covalently bound PDL (Figure 4C) compared to adsorbed PDL conditions. In addition, the neural progenitor marker, nestin, showed the opposite trend as expected to occur if a greater fraction of cells have differentiated into post-mitotic neurons. Differences in neuronal morphology were apparent with more extensive growth of projections in covalently bound cultures in closed multi-compartment devices (Figure 4D).

We next examined synapse maturation in multi-compartment devices in which PDL was covalently bound or adsorbed. We first quantified the number of synapses per βTubIII area in closed multi-compartment devices and found that synapse density was significantly greater in devices with covalently bound PDL compared to their adsorbed PDL countertypes (Figure 4E). We also found the same trend for open compartment devices (Figure 4E). These data confirm that covalently bonded PDL substrates provide a more stable surface thus providing favorable growth and maturation conditions. Further, we maintained the neurons within the covalent PDL devices until DIV 55.

### Axonal Regeneration of hSC Neurons Within Closed Multi-compartment Devices

**Figure 5:**
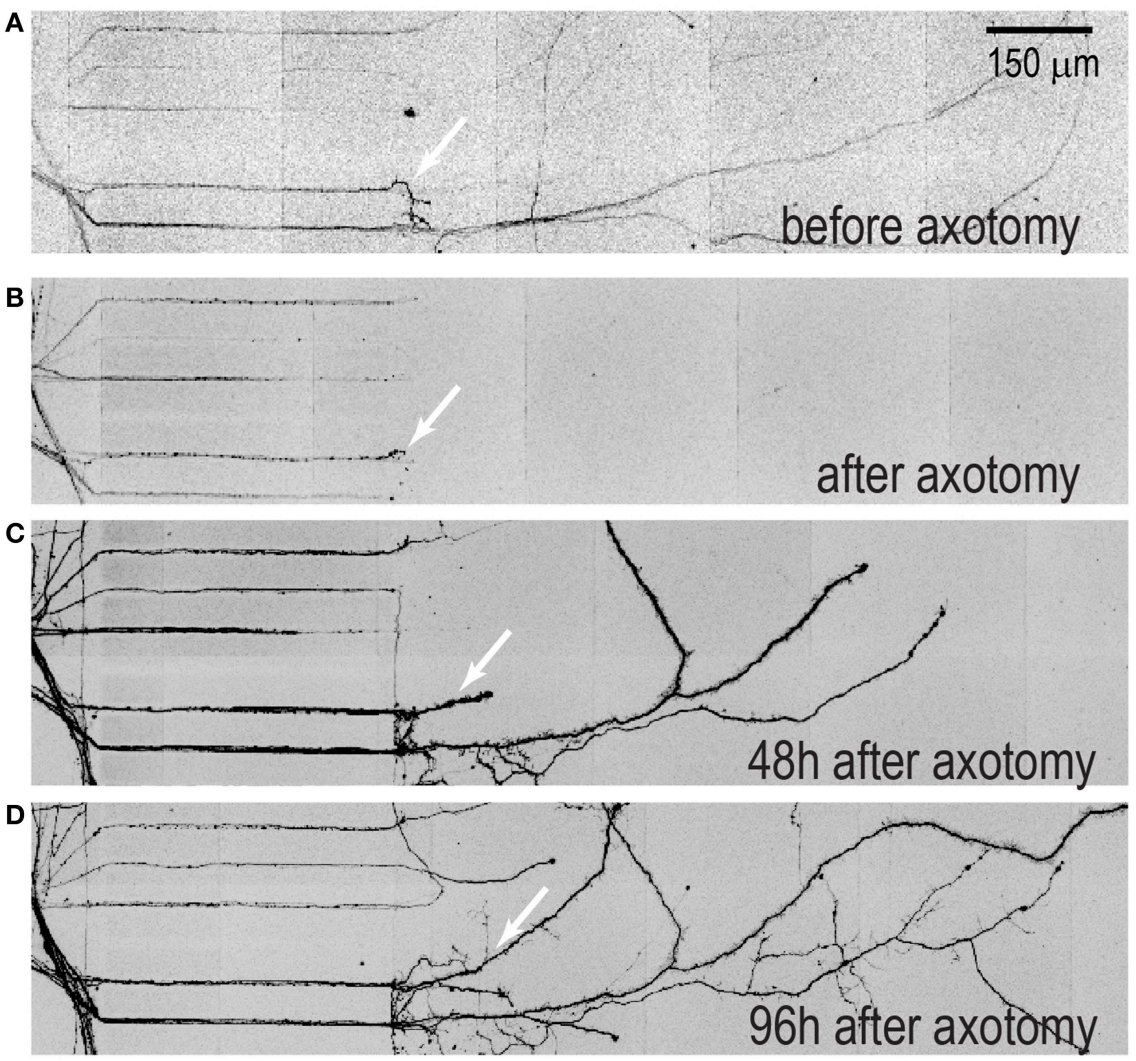
Axonal regeneration of hSC neurons within multi-compartment devices. Devices with hSC neurons were transfected with mcherry pseudotyped virus 48 h prior to axotomy to visualize neuron injury and regeneration. **(A)** A representative image of isolated axons within the axonal compartment prior to axotomy, **(B)** immediately after axotomy **(C)** 48 h after axotomy and **(D)** 96 h after axotomy. Arrows indicate regenerated axons. Scale bar, 200 μm.

Multi-compartment devices are useful for axon injury studies especially for distal axotomy due to the microgroove barrier feature, which allows axons to be removed without physically impacting the somatic compartment (Taylor et al., 2005, 2009). Here we wanted to perform a proof-of-principle experiment to determine whether the regeneration capability of injured axons from hSC neurons is similar to that observed with embryonic rodent neurons within these devices. A time series of fluorescence images of injured 50 DIV hESC derived neurons were taken before and after axotomy (Figure 5). The axons of these neurons were infected with mCherry pseudotyped virus 48 h prior to axotomy to label all neurons extending axons into the axonal compartment as performed previously (Bigler et al., 2017; Nagendran et al., 2017). Axon regeneration is visible from retraction bulbs formed after axotomy. Thus, axons from hSC derived neuron have a regenerative ability in the absence of other cells, similar to that observed in embryonic rodent neuron cultures.

### Formation of Functional Presynaptic Terminals in Multi-compartment Devices With hSC Neurons

Our group as well as others have demonstrated that micron sized beads coated with PDL, when in contact with an axon, induce presynaptic terminal differentiation including the specific accumulation of many presynaptic proteins such as bassoon, N-Cadherin, synapsin 1 and synaptophysin (Lucido et al., 2009; Taylor et al., 2013). These beads are able to mimic postsynaptic contacts thereby accelerating the formation of functional synapses. This ability for on-bead induced presynaptic accumulation has been exploited to study various mechanisms within compartmentalized devices such as local axonal translation. This would have been otherwise technically difficult, even impossible, to perform using other methods due to the presence of the larger post-synaptic compartment in standard cultures and *in vivo*.

**Figure 6:**
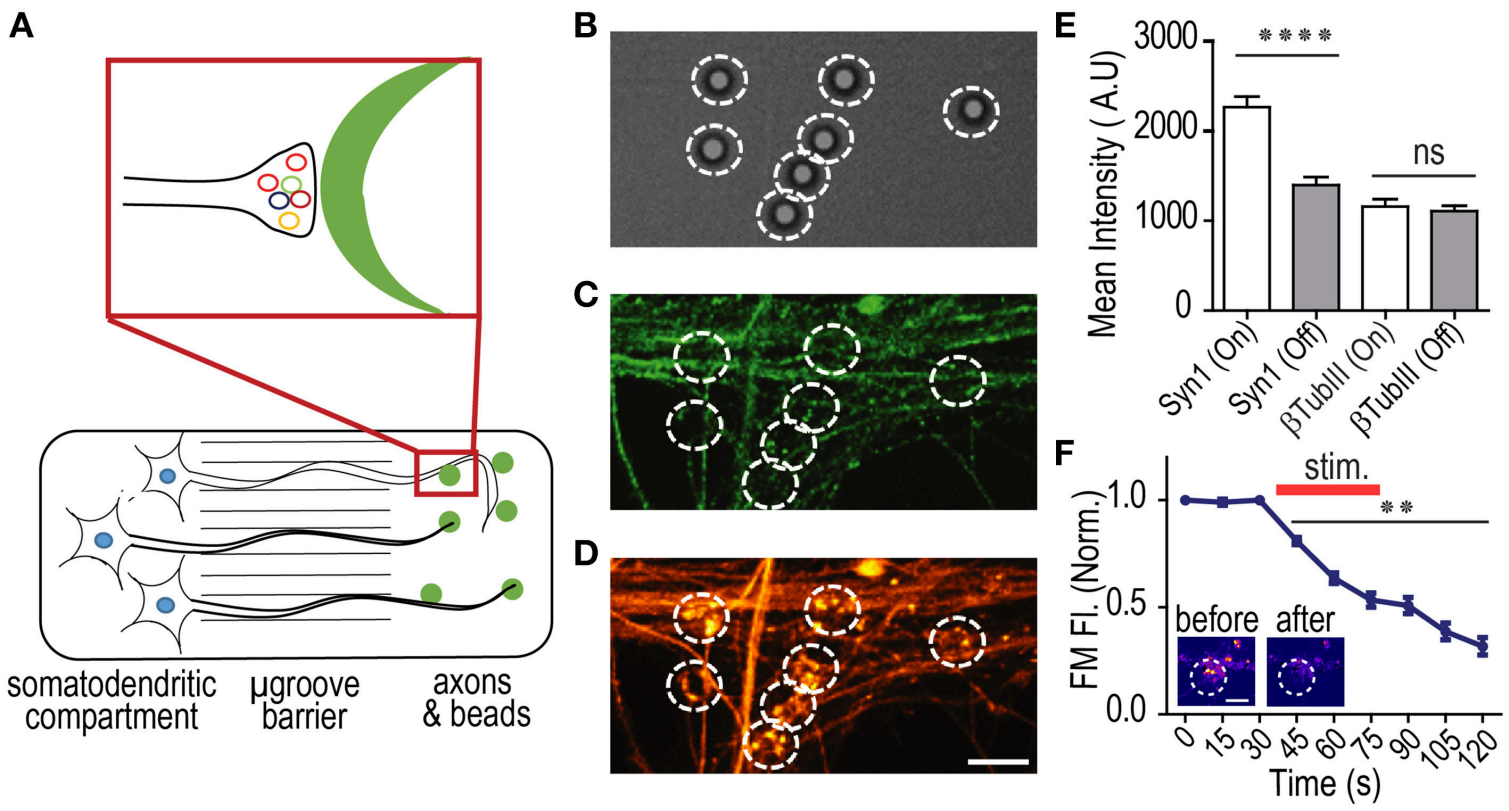
PDL coated beads induce the formation of functional presynaptic terminals in hSC neurons. **(A)** Cartoon illustration of hSC neurons in a multi-compartment device with PDL coated beads added to the axonal compartment. PDL coated beads induce presynaptic terminal formation when in contact with axons. **(B–D)** representative DIC and immunofluorescence images of β-Tubulin III (green) and Synapsin-1 (orange) within the axonal compartment of 41 DIV hSC neurons. Beads were incubated within the axonal compartment for 24 h before fixation. White circles encompass the bead ROI. Scale bar, 5 μm. **(E)** Quantification of fluorescence levels for β-Tubulin III and Synapsin-1 within ROIs surrounding beads (“on-bead”) and ROIs away from the bead (“off-bead”). *n* = 81 ROIs for all conditions; two-tailed unpaired *t*-test. **(F)** Normalized fluorescence of FM 5–95 within bead ROIs (*n* = 22 on-bead ROIs) before, during and after field stimulation to induce synaptic vesicle release, demonstrating that the presynaptic terminals formed in contact with beads undergo activity dependent vesicle release and are functional. Inset shows representative FM labeling before and after field stimulation. Scale bar, 2 μm. Two-way ANOVA, Dunnett's multiple comparisons test. ^**^p < 0.01, ^****^p < 0.0001, error bars, s.e.m.

We sought to determine whether PDL coated beads would similarly induce presynaptic terminal formation along isolated axons of hSC neurons, as described for embryonic rodent neurons (Figure 6A). We performed immunostaining for synapsin 1 on DIV 41 cultures and evaluated whether the beads induced clustering indicative of functional presynaptic terminals. As expected, our data show that synaptic vesicle clustering occurred at localized sites of bead and axon contact, supporting the formation of presynaptic terminals (Figures 6B–D). The extent of synapsin 1 accumulation was assessed by quantifying the mean fluorescence intensity of synapsin 1 within bead regions of interest (ROIs) and comparing them to axonal regions that did not contain the beads (“off-bead”). From our data, we observed that immunolabeled Synapsin 1 within the bead ROI was significantly higher in mean intensity than off-bead axonal ROIs (Figure 6E). The specificity of presynaptic protein accumulation was further confirmed by the lack of accumulation seen for the βTubIII ROIs.

Finally, we wanted to confirm that accumulated synaptic vesicles at the formed presynaptic terminals were functional. To do this, lipophilic FM dye (5–95) was used to evaluate the synaptic vesicle unloading dynamics (Harata et al., 2001; Taylor et al., 2013; Nagendran et al., 2017). FM dye was first loaded into recycling synaptic vesicles, then unloading of the dye was recorded using timelapse imaging, before, during, and after field stimulation as described and shown previously (Taylor et al., 2013; Nagendran et al., 2017). Baseline normalized FM unloading of 54 DIV hSC derived neurons showed characteristic unloading indicative of functional presynaptic terminals (Figure 6F).

## Discussion

Compartmentalized platforms for culturing neurons have a long history in neuroscience. Approaches to compartmentalizing neurons have included the use of Campenot Chambers (Campenot, 1977), filter-based isolations (Torre and Steward, 1992), and more recently microfluidic chambers. The majority of compartmentalized microfluidic chambers in use are PDMS-based and attached to polylysine coated glass (Neto et al., 2016).

Our data support the use of closed channel microfluidic chambers together with glass covalently bound with PDL for maintaining the health and maturation of long-term cultures of hSC-derived neurons. Notably, neurons still matured within open devices, although not as robustly, providing an option for experiments requiring open access to compartments. We speculate that the lower media per unit cell area within closed devices may provide a favorable microenvironment for transfer of neurotrophins or other soluble factors within the neuronal network resulting in improved synapse maturation. The increased fluid flow during media exchanges within the 400T devices compared with 100T devices did not appear to affect neuronal differentiation or synapse maturation.

Previous studies have primarily used multi-compartment microfluidic devices for the culture of *immature* hSC-derived neurons. Lee et al., investigated differentiation potential of hSC-derived neurons in a poly(dimethylsiloxane) (PDMS) based multi-compartment device with microgrooves for axon guidance. They demonstrated that hSC-derived neurospheres were able to differentiate into neurons and form neural networks within the device. In addition, they observed that migration was dependent on the stage of differentiation as neurons tended to migrate toward the microgrooves as opposed to neuroprogenitor cells (Lee et al., 2014). (Kerman et al., 2015) found it difficult to use conventional cell culture platform for myelination studies relating to stem cell derived neurons because neurites and cell bodies were intertwined; instead, they used a multi-compartment device to achieve axon isolated area. These studies support the significant utility of compartmentalized devices for hSC-derived neuron research. However, the devices only maintained SC derived neurons for shorter periods of about 5 days *in vitro* (DIV) to 14 DIV.

Lastly, we demonstrated the use of the multi-compartment devices for two proof of principle experiments using hSC-derived neurons. Axon injury and regeneration experiments with retrograde labeled neurons clearly show the ability of these neurons to intrinsically regenerate in the absence of inhibitory cues. In addition, target mimics (PDL-coated beads) applied to isolated axons induced functional presynaptic terminal formation, as similarly found in murine cultures (Taylor et al., 2013; Pinto et al., 2016). Together, these results provide further evidence of the utility of this platform for neurobiological experimentation using hSC-derived neurons.

## Author Contributions

JK acquired and analyzed data and wrote the manuscript. TN instructed viral transduction, injury/regeneration, immunostaining, and FM experiments and helped write the methods section. JH provided open microfluidic chambers. AT analyzed data and wrote the manuscript.

### Conflict of Interest Statement

The authors declare that this study received funding from Xona Microfluidics, LLC as part of NIH awarded STTR grants (R42 MH097377, R41 NS108895). JH and AT declare competing interests. AT is an owner and Chief scientist of Xona Microfluidics, LLC, and an inventor of the multi-compartment microfluidic device (US 7419822 B2, EPO 1581612, EPO 2719756). JH is an owner and Chief Operating Officer of Xona Microfluidics, LLC. The remaining authors declare that the research was conducted in the absence of any commercial or financial relationships that could be construed as a potential conflict of interest.
